# Patient preferences for outcomes in critical care trials (OPTICS): preliminary results

**DOI:** 10.1186/cc14628

**Published:** 2015-03-16

**Authors:** J Muscedere, F Lamontagne, G Boyd, M Herridge, S Fleury, T Sinuff

**Affiliations:** 1 Queen's University, Kingston, ON, Canada; 2 University of Sherbrooke, QC, Canada; 3 University of Toronto, ON, Canada

## Introduction

Although patient-centered outcomes are important to inform therapeutic choices for critically ill patients, patient preferences for outcomes in critical care studies are unknown. It is also unknown whether outcome preferences differ between researchers, decision-makers and those who have never been critically ill (citizens). The aim of the OPTICS Program is to investigate these preferences. Herein we report the preliminary results for outcomes preferences in citizens.

## Methods

We recruited and surveyed lay public members without a history of critical illness as to their preferences for outcomes in critical care trials. After an in-person educational session, citizens were asked to rank 11 potential critical care trial outcomes in order of personal preference. Each outcome was also rated for importance on a 7-point Likert scale. Participants were then asked to indicate their agreement with potential tradeoffs between potential outcomes.

## Results

The in-person session was attended by 31 citizens whose had a mean age (SD) of 71.6 (5.9) years and 1.5 (1.4) chronic health conditions; 25 (81%) had partially or fully completed post-secondary school education. Of the 11 potential outcomes, the three outcomes ranked of highest importance were: permanent brain dysfunction, quality of life, and requirement for long-term institutional care. The three outcomes ranked of least importance were: duration of hospitalization, death after a prolonged illness, and occurrence of delirium. When rated on a 7-point Likert scale the results were similar. Of the participants, 24/27 (89%) indicated that they would be willing to receive a therapy which was associated with a higher mortality rate but resulted in an improved quality of life in survivors. Conversely, 16/27 (59%) indicated that they would not be willing to receive a therapy which increased the chances of survival but was associated with a reduced quality of life in the survivors.

## Conclusion

The citizens surveyed valued outcomes associated with quality of life and intact neurological function. Mortality and other indices of duration of critical illness were valued less. These preferences need to be confirmed in larger groups with greater diversity. Future research also needs to further understand these outcome preferences in survivors of critical illness, clinicians, decision-makers, and researchers. Understanding the outcome preferences of pertinent stakeholders and whether these preferences are congruent is imperative to inform the design of future critical care trials.

